# At mid- to long-term follow-up after proximal hamstring tendon avulsion; there was greater fatty infiltration, muscle atrophy and strength deficit in the hamstring muscles of the injured leg than in the uninjured leg

**DOI:** 10.1186/s13018-023-03582-2

**Published:** 2023-02-16

**Authors:** Elsa Pihl, Mikael Skorpil, Olof Sköldenberg, Carl Johan Hedbeck, Kenneth B. Jonsson

**Affiliations:** 1 Unit of Orthopeadics, Department of Clinical Sciences at Danderyds Hospital, Karolinska Institutet, Ortopedmottagningen Danderyds Sjukhus, 182 88 Stockholm, Sweden; 2 Danderyd University Hospital Corp, Stockholm, Sweden; 3 Department of Molecular Medicine and Surgery, Karolinska Institutet, Stockholm, Sweden; 4 Department of Surgical Sciences, Uppsala University Hospital, Uppsala, Sweden

**Keywords:** Magnetic resonance imaging, Proximal Hamstring avulsion, Muscle quality, Fatty infiltration, Strength

## Abstract

**Background:**

Proximal hamstring tendon avulsions (PHAs) may be treated nonoperatively or operatively. Little is known about the result of the injury, and its treatment, on the quality and function of the hamstring muscle after healing and rehabilitation. We hypothesized that the injured leg would have greater fatty infiltration and atrophy than the uninjured leg at follow-up and that these findings would correlate to muscle weakness.

**Methods:**

In a cross-sectional cohort study, 48 patients treated for PHA, either operatively or nonoperatively, were re-examined 2–11 years post-treatment. We measured muscle strength with isokinetic strength tests, and muscle volume and fatty infiltration with MRI.

Primary outcomes were hamstring muscle quality, quantified by outlining the cross-sectional area slice-by-slice, and the degree of fatty infiltration estimated using the Goutallier grading method. Secondary outcome was concentric isokinetic hamstring muscle strength measured using BioDex at 60°/sec and tendon attachment assessed on MRI. Comparisons with the outcomes of the uninjured leg were made.

**Results:**

The total hamstring muscle volume was on average reduced by 9% (SD ± 11%, *p* < 0.001) compared to that of the uninjured leg. Fatty infiltration was significantly more severe in the injured hamstrings than in the uninjured hamstrings (*p* < 0.001). This was also true when only analyzing operatively treated patients. The reduction in muscle volume and increase in fatty infiltration correlated significantly (*r* = 0.357, *p* = 0.013), and there was also a statistically significant correlation with muscle atrophy and reduction in isokinetic strength (*r* = 494, *p* < 0.001).

**Conclusion:**

PHA injuries result in fatty infiltration and muscle atrophy and the muscle quality impairment correlates with residual muscle weakness.

## Key points

- Proximal hamstring avulsion results in muscle atrophy and fatty infiltration.
- The muscle degeneration after proximal hamstring avulsion is correlated to reduction in muscle strength.
- The muscle degeneration and its correlation to reduction in muscle strength hold true also for operatively treated patients.

## Introduction

### Background

Hamstring muscles injuries have been reported to represent a large amount of al all soft tissue sporting injuries [1]. Hamstring injuries may be divided by which part of the muscle is affected. Proximal and distal tendon injuries are less common than the frequent muscle belly injury [2, 3]. A rare variant of hamstring injury is the Proximal Hamstring Avulsion (PHA) with an incidence of 3–10% in the elite athlete population [4]. PHA seems predominantly to affect middle-aged, non-elite athlete patients and degenerative change in the tendon likely plays a role in the pathogenesis [5, 6]. The injury occurs by accidentally slipping or rapidly lengthening the muscle during an eccentric contraction, e.g., during waterskiing. In PHA injuries, the two hamstring muscle tendons (Semimembranosus and the conjoint tendon of the Semitendinosus and Biceps femoris) are commonly avulsed from the ischial tuberosity together but on rare occasions, only the conjoint tendon is affected [3, 7, 8].

The treatment options for proximal hamstring tendon avulsions (PHAs) are either surgical reattachment of the tendons followed by rehabilitation or nonoperative treatment with rehabilitation only. The theoretical reasoning for surgical reattachment of the tendons to their footprint is that restoration of muscle biomechanics should protect muscles from fatty infiltration, atrophy and loss of strength [9, 10]. Studies have found muscle strength to be reduced by 10–45% compared to the uninjured side [9–16].

Magnetic resonance imaging (MRI) or computerized tomography (CT) can be used to measure muscle atrophy and to estimate fatty infiltration of muscles [17–20]. No previous study of PHA have measured muscle volume or the correlation of imaging outcomes to strength.

Recently, van der Made et al. quantified fatty infiltration of the injured hamstrings muscle in 59 PHA patients using the Goutallier grading system [21]. MRI images acquired one year after injury did not demonstrate a difference in fatty infiltration between patients treated operatively and those treated nonoperatively [21]. Fatty infiltration using the Goutallier grading system has also been described by others evaluating PHA patients and their muscle quality at follow-up [10].

In this study, our primary goal was to quantify muscle atrophy and fatty infiltration of the hamstring muscles in patients treated for PHA regardless of treatment modality, and our secondary goal was to determine whether the loss of muscle volume and increase in fatty infiltration correlate with the isokinetic strength of the hamstring muscles. Additionally, we aimed at carefully examining the morphology and attachment of the proximal hamstring tendons.

We hypothesized that the injured leg would have more fatty infiltration and atrophy than the uninjured leg at follow-up and that these findings would correlate to muscle weakness.

## Methods

### Study design and setting

A single-center cross-sectional cohort study was performed at an academic university care center between September 2018 and May 2019.

### Participants

A consecutive series of all patients diagnosed and treated, either operatively or nonoperatively, for PHA at Danderyd Hospital from 2007 to 2016 were identified from the radiology administrative system and the surgical planning system. We have previously presented parts of the same cohort of patients, their treatment and rehabilitation regime, as well as patient reported outcomes, and physical performance-based tests at long-term follow-up have been analyzed and presented [15, 22].

#### Inclusion and exclusion criteria

The inclusion criterion was patients treated for PHA with at least the conjoint tendon of biceps femoris long head and semitendinosus or the semimembranosus tendon avulsed from the ischial tuberosity.

The exclusion criteria were bilateral PHA and other major lower extremity injury or disease with sequelae unrelated to the PHA. Additionally, patients with severe obesity [body mass index, ≥ 35] at the time of follow-up, claustrophobia or documented re-rupture were excluded.

### Data collection

Baseline data were collected from medical records and included patient characteristics, injury mechanism, MRI report at diagnosis, time to treatment, treatment modality and adverse events. At follow-up, we measured imaging variables and muscle strength variables. It was time consuming for patients to participate in the study, for their convenience, the MRI was performed the same day after the strength tests. In some cases, the MRI was performed some days before the strength tests or some days or weeks later. Recruitment strategy for this cohort is previously published [15]. Study data were collected and managed using Research Electronic Data Capture (REDCap) electronic data capture tools hosted at Karolinska Institute [23].

### Magnetic resonance imaging

MRI scans were performed on a Philips Ingenia 3 T system. Patients were examined in the supine position. Imaging sequences included axial and coronal PD-weighted, as well as axial and coronal PD-weighted, SPAIR (spectral adiabatic inversion recovery) of both proximal thighs. Additional images were axial T1-weighted images of both entire thighs, as well as sagittal T2-weighted and sagittal STIR of the injured thigh.

### Variables

Primary outcomes were total hamstring muscle volume and quality as quantified degree of fatty infiltration. Secondary outcomes were isokinetic hamstring muscle strength and tendon attachment.

#### Imaging-fatty infiltration

**Fig. 1:**
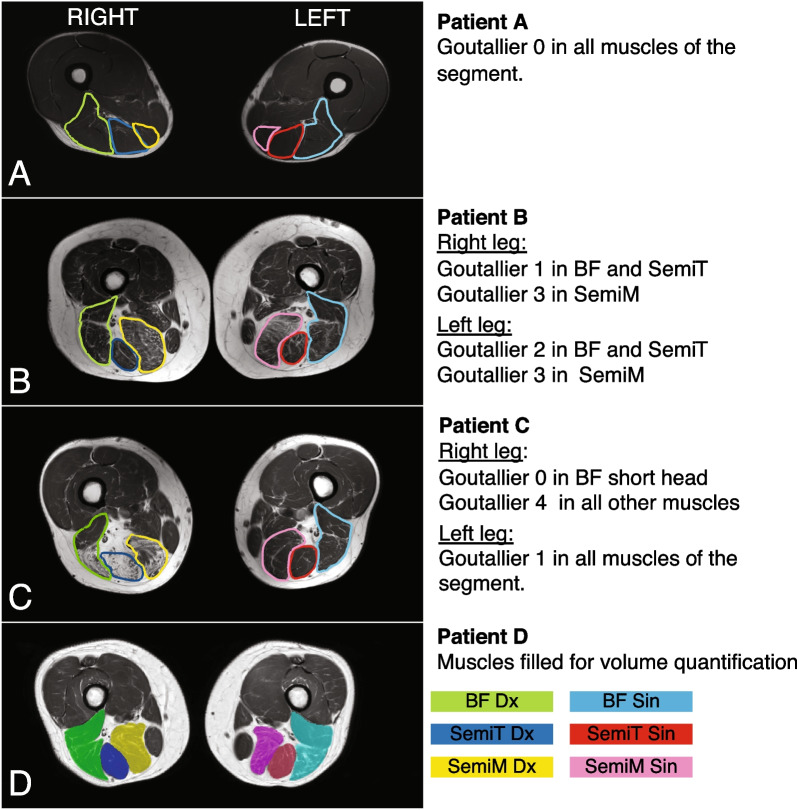
Depiction of the Goutallier scores of three different patients (**A**, **B**, and **C**). Nota bene: In patient C, the right leg of the Biceps femoris short head has Goutallier 0 and long head 4. Panel D demonstrates the outline of the cross-sectional area for muscle volume quantification

The fatty infiltration of the muscles was assessed according to the Goutallier grading system [17]. This classification was developed for the rotator cuff muscles using CT, but was later transferred to T1-weighted MRI images [18]. In this system, cross-sectional images are graded on an ordinal scale as follows: 0, normal muscle; 1, some fatty streaks; 2, less fat than muscle; 3, fatty infiltration of 50%; 4, fatty infiltration of more than 50% (see Fig. 1 for examples) This classification has previously been used examining patients after PHA [10, 21].

The classification was performed using axial T1 weighted images in the open-source software Horos© version 3.3.6. by visually inspecting each hamstring muscle separately. To get as much information from the images as possible, a modification of the original classification was performed: All the segments of the muscle were inspected, and a Goutallier score representing the entire muscle was set. The classification was performed by an orthopedic surgeon (EP). To obtain reliability measures, the classification was performed twice on every image, June 2020 and September 2021. In cases where there was a discrepancy between the first and second scores, an expert opinion by a consultant in radiology (MS) was obtained to reach a final classification decision. The total Goutallier score for the injured and uninjured thighs was calculated by adding the Goutallier scores of the three separate muscles.

#### Imaging-muscle volume

Muscle volume was measured in cm^3^ by slice-by-slice segmentation [20]. The anonymized Digital Imaging and Communications in Medicine (DICOM) series obtained from the MRI camera were fused together and converted into VisualizationToolKit (VTK) using MATLAB version R2018a. The open-source software ITK-snap, version 3.8.0 (itksnap.org), was used for segmentation of the VTK files [24]. Muscle segmentations were performed by one orthopedic surgeon (EP) and reviewed by a consultant in radiology (MS). Muscle areas were outlined slice wise on an axial T1 image. The volume of the binary segmentation was calculated by ITK-snap taking the total number of voxels segmented times the volume of each voxel. To calculate reliability measures, the semitendinosus muscle was outlined twice for every second patient. Agreement was defined as repeated measures within 1 cm^3^.

#### Imaging-tendon attachment

A consultant in radiology (MS) assessed whether the tendons were attached to the ischial tuberosity. A surgically reattached tendon in continuity to the ischial tuberosity and without any high signal on PD-weighted SPAIR at the ischial tuberosity was classified as totally attached. If there was some high signal on PD-weighted SPAIR at the attachment, it was classified as partially attached. If the tendon was not in continuity with the ischial tuberosity or retracted, it was classified as nonattached. Tendons not operated on were classified as totally attached if all tendon fibers were in continuity to the ischial tuberosity and partially attached if some of the tendon fibers were in continuity. If no tendon fibers were in continuity with the ischial tuberosity or retracted, they were classified as nonattached.

#### Muscle strength

Concentric isokinetic muscle strength was assessed with peak torque at 60°/sec measured with the Biodex Pro System 4 dynamometer (Biodex Medical Systems Inc., Shirley, New York, USA). A description of the measurement procedure and accuracy was already published [15] in summary: Patients were positioned following a predetermined protocol, allowing for individual adjustment. Structured verbal encouragement was used, and the noninjured leg was tested before the injured leg.

The resting period between each test was approximately two minutes. The patient was seated in upright position, with the lateral femoral condyle in the rotation axis and the range of motion was set to 0–90 degrees. The peak torque in knee flexion was tested at 60°/sec during five maximum concentric repetitions. The highest observed torque in Newtons (Nm) was recorded.

### Statistics

Demographic variables were described by the mean and standard deviation (SD) and continuous and categorical variables were described by count and percentage. Confidence intervals (CI) at 95% confidence level were used. Test–retest reliability for imaging outcome measures was calculated using the intraclass correlation coefficient (ICC). For comparison of means of the injured vs. uninjured leg, the paired t test was used for normally distributed variables and the Wilcoxon signed rank test for nonnormally distributed variables.

Correlations were analyzed with the Spearman correlation coefficient. Cutoff values for the strength of correlations were *r* =  >  ± 0.7 (strong), *r* =  ± 0.5–0.7 (moderate), *r* ±  < 0.5–0.3 (weak) and *r* ≤ 0.3 (negligible) [25], and the level of significance was set at *p* < 0.05. Subgroup analyses were made on patients treated operatively and on patients with tendon avulsion > 2 cm.

No separate power calculation was performed prior to this descriptive study, and no imputations were performed for missing data. Complete case analysis was used in all analyses. Statistical analysis was performed using IBM SPSS statistics version 28 for Mac.

## Results

### Participants and baseline demographics

**Fig. 2:**
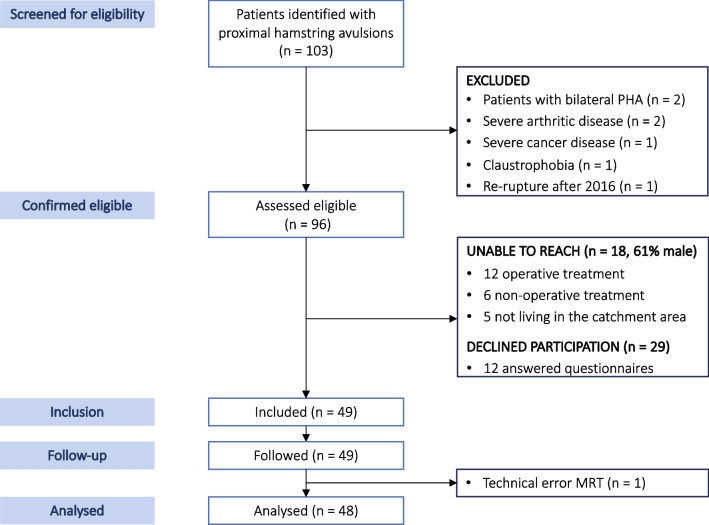
Study flowchart

We identified 96 eligible patients and included 49 patients, 48 of whom provided data for the final analysis (Fig. 2), with a follow-up time of 2–11 years (mean 5.6 (SD ± 2.7)). Seventy-five percent of the included patients were treated operatively. Seventy-three percent had all tendons avulsed > 2 cm at injury and mean time to treatment was 17.6 (SD ± 14.1) days. (Table 1).

**Table 1:** Baseline data

	n (%)	Mean (±SD)
Sex, female	22 (45.8)	
Age at injury, years		50.8 (9.7)
*Activity at injury*		
Sporting injury		30 (62.5)
Vehicle accident		2 (4.2)
Slip or fall		10 (20.8)
Other		6 (12.5)
MRI Finding at diagnosis**		
Incomplete, two tendons avulsed	8 (16.7)	
Complete, three tendons avulsed < 2 cm	5 (10.4)	
Complete, three tendons avulsed > 2 cm	35 (72, 9	
Time to treatment, days		17.6 (14.1)
Operative		18.3 (15.1)
Nonoperative		15.5 (10.8)
*Treatment*		
Operative	36 (75)	
Nonoperative	12 (25)	
Follow-up time, years		5.6 (2.7)

**MRI finding according to the radiology report signed by two consultants diology

### Fatty infiltration

**Fig. 3:**
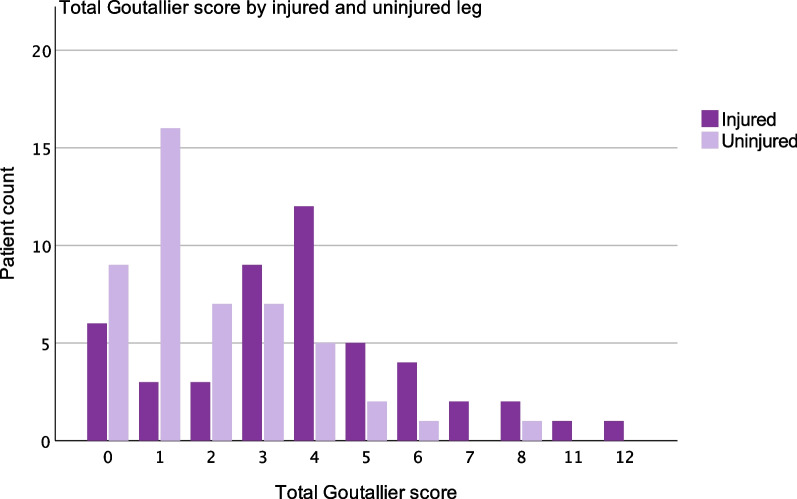
Patient count of each total Goutallier score for injured and uninjured legs. The distribution between the thighs differs, with more patients having higher scores in the injured leg than in the uninjured leg.

We found a significantly greater total fatty infiltration (*p* < 0.001) in the injured hamstring muscle group than in the uninjured hamstring muscle group (Table 2 and Fig. 3). The median total Goutallier score of the injured hamstrings was 4 (interquartile range (IQR) 3–5) compared to 1 for the uninjured side (IQR 1–3) (Fig. 3). The distribution of Goutallier scores was significantly different between the extremities for each of the three hamstring muscles (*p* < 0.001) (Table 2). The Goutallier classification exhibited high intra-rater reproducibility with an ICC of 0.82–0.91 for every hamstring muscle separately.

**Table 2:** Goutallier score of separate hamstring muscles at follow-up

	Biceps femoris		Semitendinosus		Semimembranosus	
	Injured leg	Uninjured leg	Injured leg	Uninjured leg	Injured leg	Uninjured leg
Goutallier score	*n* (%)	*n* (%)	*n* (%)	*n* (%)	*n* (%)	*n* (%)
0	9 (19)	26 (54)	12 (25)	33 (69)	9 (19)	13 (27)
1	24 (50)	19 (40)	25 (54)	13 (27)	13 (27)	24 (50)
2	13 (27)	2 (4)	6 (13)	2 (4)	18 (38)	8 (17)
3	0 (0)	1 (2)	2 (4)	0 (0)	4 (8)	3 (6)
4	2 (4)	0 (0)	2 (4)	0 (0)	4 (8)	0 (0)
Total	48 (100)	48 (100)	48 (100)	48 (100)	48 (100)	48 (100)
*p* value	< 0.001		< 0.001		< 0.001

### Muscle volume

**Fig. 4:**
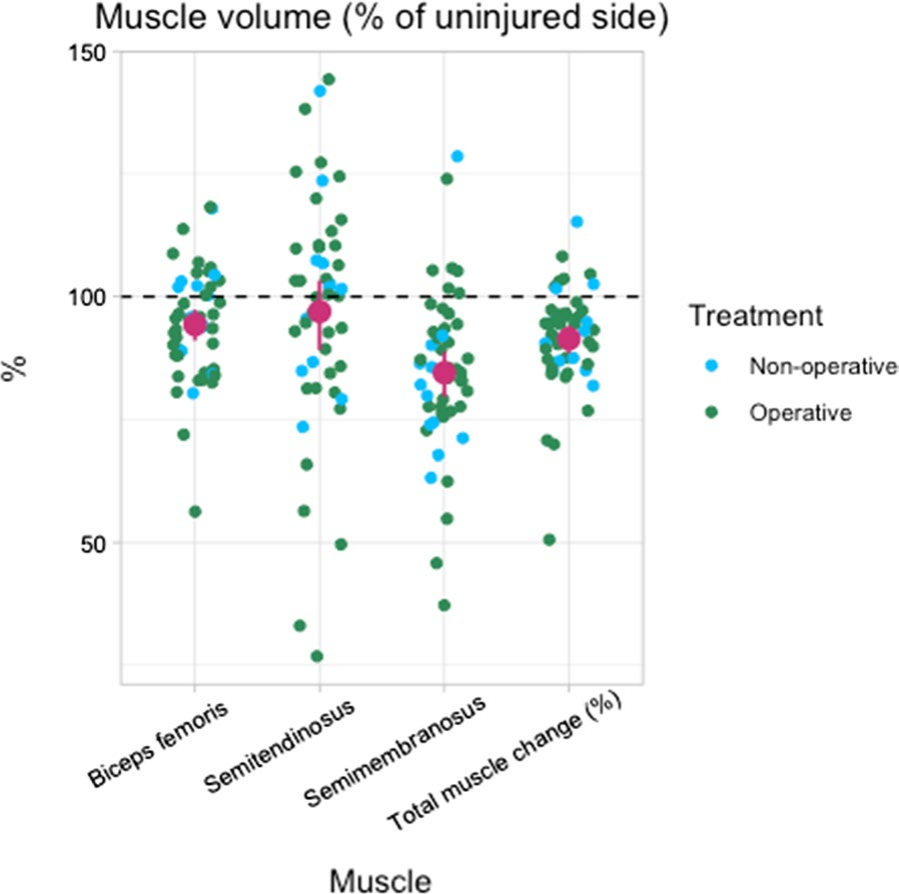
Distribution of patient (operative and nonoperative treated) muscle volume as a percentage of the uninjured side of the three different muscles and the total muscle volume. Purple dots represent the mean muscle volume loss and CI 95%. The semimembranosus muscle shows the greatest muscle volume loss

The mean total volume of the injured hamstring muscle group was 1354 cm3 (± SD 405) compared to 1506 cm3 (± SD 480) of the uninjured hamstrings, representing a mean muscle volume deficit of 9% (± SD 11%). The semimembranosus muscle exhibited the greatest mean deficit in muscle volume (16% ± SD 17%) (Fig. 4). The muscle volume measurements exhibited high reproducibility with an ICC of 0.99.

### Tendon attachment

**Fig. 5:**
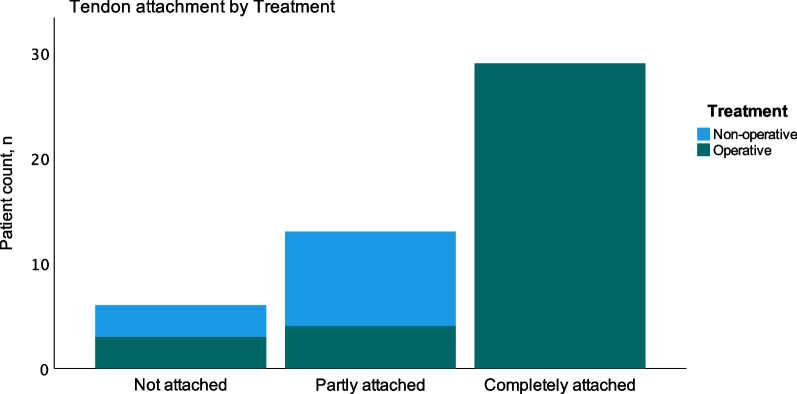
Distribution of different tendon attachments separated by treatment. Only operationally treated patients had the tendons completely attached

When examining the hamstring insertion site at follow-up, we found that 29/48 of patients had proximal hamstring tendons that were completely attached to the ischial tuberosity, 13/48 had tendons that were partly attached, and 6/48 had tendons that were completely detached. Among operatively treated patients, most tendons were completely attached (Fig. 5), whereas none of the nonoperatively treated patients had tendons that were completely attached.

We carefully examined the morphology of the proximal hamstring tendons of the nonoperatively treated patients. In three patients, there were no hamstring tendons in continuity with the tuber ischii. In these cases, the semimembranosus and conjoined tendons healed together with minor fibrosis, and in two cases, the fibrosis also involved the fascia of the external obturator muscle. The fibrosis mostly continued to the ischial tuberosity and could be difficult to distinguish from few remaining tendon fibers.

In eight non-operated patients, the entire or part of the semimembranosus tendon was in continuity with the ischial tuberosity, while the conjoined tendon was not. The conjoined and the semimembranosus tendon had healed together with fibrosis, either proximally or elongated. The amount of fibrosis differed, and in one case, it involved the fasciae of the external obturator and the adductor magnus muscles, and in another case, it had healed structurally to resemble a neotendon.

The last case had a partially healed, thickened semimembranosus tendon with the conjoined tendon in continuity with the tuber ischii.

### Strength tests

Forty-two patients were able to perform the peak torque test. The mean peak torque of the injured hamstrings was 60 Nm (± SD 24) compared to 66 Nm (± SD 23) of the uninjured hamstrings. The mean deficit was 9% (± SD 22%, *p* < 0.008).

### Correlations

**Fig. 6:**
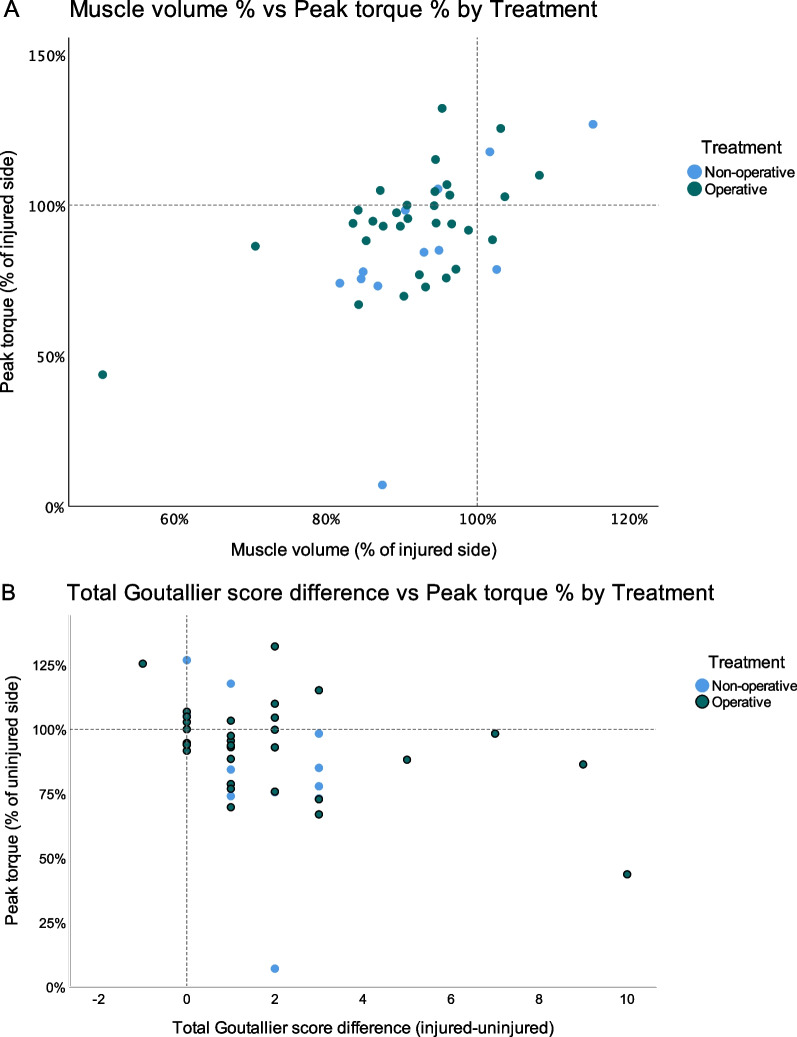
(**A**) Scatterplot of muscle volume (% of uninjured side) vs. peak torque (% of injured side). Lines displayed at 100% represent no difference between the legs. (**B**) Difference in total Goutallier between the legs by peak torque (% of uninjured side). Lines indicate where there was no difference between the legs

When analyzing the correlations of muscle volume deficit and difference in total Goutallier score between injured and uninjured hamstrings with peak torque, we found statistically significant weak correlations (*r* = 0.357–0.494, *p* < 0.001–0.05) (Fig. 6).

### Subgroup analysis

In a subgroup analysis of only operatively treated patients, the overall results were similar (Figs. 4 and 6); the muscle volume deficit of the injured leg was 9% (± SD 11%, *p* < 0.001), with the greatest loss of the semimembranosus 15% (± SD 17%), and the total Goutallier score was significantly different between the legs (*p* < 0.001). The peak torque deficit between the legs was 7% (± 17%, *p* = 0.07).

When limiting the analysis to the 35 patients (30 operative and 5 nonoperative treated) with a total avulsion > 2 cm, the differences in fatty infiltration and muscle volume remained.

## Discussion

The most important finding of this study was that patients, several years after acute PHA, have more extensive fatty infiltration, muscle atrophy and loss of isokinetic strength in the injured hamstrings than in the uninjured hamstrings. Even though the study was not designed to compare operated and non-operated patients, similar results were obtained when the analysis was limited to patients treated operatively, suggesting that surgery cannot protect against muscle degeneration. This is consistent with previous studies of tendon avulsions [19, 26–28]. However, a prospective randomized trial is needed to quantify the potential benefits of operative treatment in a scientifically sound manner.

In our data, there were differences in volume loss between the muscles. The semimembranosus muscle had the greatest volume loss both in the main and subgroup analyses, suggesting differences in recovery of the different hamstring muscles, even when all tendons are avulsed > 2 cm or if surgically reattached. Interestingly, differences in recovery between muscles have been observed after rotator cuff repairs [29], and compensatory muscle hypertrophy may contribute to strength variability after Achilles tendon repair [19]. Also, recovery differences of the hamstring muscles have previous been shown. In one study on subjects affected by hamstring muscle strains in the long head of biceps femoris, atrophy of that muscle was often accompanied by hypertrophy of the biceps femoris short head [30]. We found that the distribution of muscle volume in relation to the uninjured side is especially large in the semitendinosus muscle, suggesting that some patients may compensate for the loss of function of the semimembranosus with hypertrophy of this muscle. The difference in recovery between the muscles can support the reasoning of Lempainen et al. that each hamstring muscle has its own function and anatomy, and therefore should be considered individually already at the injury stage [3]. However, when interpreting this observation, it needs to be understood that the slice-by-slice technique measures the entire muscle volume, regardless of whether it is infiltrated by fat or not.

Our study expands on previous studies that examined muscle status by MRI after PHA by demonstrating loss of muscle volume in addition to fatty infiltration even when the follow-up time is several years [10, 21]. Moreover, our results support, with low correlations (0.4–0.5 (*p* < 0.001–0.05)), that muscle volume deficit and greater fatty infiltration in the injured leg result in a strength deficit. This is consistent with previous studies of the hamstrings where eccentric strength has been shown to be correlated to biceps femoris muscle volume [31] and with similar findings in other tendon injuries. For example, one study of Achilles tendon ruptures found loss of muscle volume (11–13%), greater fatty infiltration and correlation to strength deficit (12–18%) in the affected leg at follow-up [19].

We previously published a study on the same cohort of patients showing that two patient reported outcome measures, the Lower Extremity Functional Scale (LEFS) and Perth Hamstring Assessment Tool (PHAT), correlated significant to isokinetic strength (*r* = 0.307 and 0.340), indicating that the strength deficit measured have subjective relevance to the patients. Together with the current study, we have now investigated the complete chain of causality between the injury and patient reported outcomes. We have established the effect of the injury on muscle morphology, muscle strength, physical function and patient reported outcomes.

This study also contributes information on the healing of proximal hamstring tendons when they are not treated with surgical reattachment. None of the patients treated nonoperatively had a complete attachment of the tendons to their footprint, but nine patients had some continuity to the footprint and only three none. The only previous study indicated a lower ratio of continuity but reported the formation of a neotendon in many cases [21]. In the current cohort, this was seen only in one patient. Additionally, looking at operatively treated patients, our results, combined with previous publications on both PHA and other tendon injuries, suggest that surgical reattachment of an avulsed tendon cannot guarantee tendon continuity at long-term follow-up [21, 27, 28]. Reruptures or incomplete healing of surgically attached tendons are likely to contribute to the variability in muscle quality and strength after rehabilitation.

## Strength

This is the first study with mid- to long-term follow-up, examining muscle volume and correlation of imaging outcomes to strength in both operatively and nonoperatively treated PHA. We examined a relatively large cohort of PHA patients and used the best available measures to evaluate muscle quality (MRI) and concentric strength (isokinetic test) [16]. Our different imaging measures have high intra-rater reliability (ICC 0.81–0.99), which is consistent with previously published reliability measures, for the Goutallier score [32] and for slice-by-slice volume measures [20, 33]. The isokinetic test used is the gold standard of strength measurements, and peak torque at 60°/s has been shown to be the most discriminatory test in detecting strength differences between the injured and uninjured extremity after PHA [16].

## Limitations

One limitation of our study is that the Goutallier score is an individual assessment of the visual impression of an image. The score has been used in other PHA studies [10, 21], but is not validated for the hamstring muscles. When performing the study, we did not have access to an MRI protocol for quantitative analysis of fatty infiltration, such as Dixon [34]. Moreover, with the cross-sectional study design, we cannot draw conclusions on the reasons for the loss of strength and muscle quality. For example, we do not have data on muscle quality and strength at the time of injury, which means that we do not know if the fatty infiltration, muscle volume and strength deficit were already present prior to the injury. The treatment choice in our cohort is not random and is likely affected by bias by indication. For this reason, the study design does not allow for direct analysis of the effect of treatment on these outcome parameters. Instead, we tested the validity of our main results in subgroup analyses of patients treated operatively and of patients having all tendons avulsed > 2 cm at injury. In these analyses, the difference between the injured and uninjured hamstrings remained.

Our study was also limited by the fact that we cannot sufficiently control for or further analyze three important confounders: a more detailed classification of the injury, the quality of the surgical procedure and the intensity of the patient’s rehabilitation and muscle strengthening exercises.

## Conclusion

At an average of 6 years after acute proximal hamstring avulsions, patients had greater fatty infiltration and muscle atrophy in the affected hamstrings than in the uninjured leg. The muscle quality impairment significantly correlated with the degree of the strength deficit of the injured leg. The same results were obtained when the analysis was limited to patients who had been treated operatively.

## Data Availability

The datasets generated and/or analyzed during the current study are not publicly available due to integrity of the participants but are available from the corresponding author (EP) upon reasonable request.

## Abbreviations

CI: Confidence intervals
CT: Computer Tomography
DICOM: Digital Imaging and Communications in Medicine
ICC: Intraclass Correlation Coefficient
LEFS: Lower Extremity Functional Scale
MRI: Magnetic Resonance Imaging
PHA: Proximal hamstring avulsions
PHAT: Perth Hamstring Assessment Tool
PROM: Patient-Related Outcome Measure
SD: Standard Deviation
VTK: Visualization Tool Kit

## References

[1] Silvers-Granelli HJ, Cohen M, Espregueira-Mendes J, Mandelbaum B (2021). Hamstring muscle injury in the athlete: state of the art. J isakos.

[2] Entwisle T, Ling Y, Splatt A, Brukner P, Connell D (2017). Distal Musculotendinous T Junction Injuries of the Biceps Femoris: an MRI Case Review. Orthop J Sports Med.

[3] Lempainen L, Kosola J, Pruna R, Sinikumpu JJ, Valle X, Heinonen O (2021). Tears of biceps femoris, semimembranosus, and semitendinosus are not equal-a new individual muscle-tendon concept in athletes. Scand J Surg.

[4] van der Made AD, Reurink G, Gouttebarge V, Tol JL, Kerkhoffs GM (2015). Outcome after surgical repair of proximal hamstring avulsions: a systematic review. Am J Sports Med.

[5] Irger M, Willinger L, Lacheta L, Pogorzelski J, Imhoff AB, Feucht MJ (2019). Proximal hamstring tendon avulsion injuries occur predominately in middle-aged patients with distinct gender differences: epidemiologic analysis of 263 surgically treated cases. Knee Surg Sports Traumatol Arthrosc.

[6] Bodendorfer BM, Curley AJ, Kotler JA, Ryan JM, Jejurikar NS, Kumar A (2017). Outcomes after operative and nonoperative treatment of proximal hamstring avulsions: a systematic review and meta-analysis. The Am J Sports Med.

[7] Ahmad CS, Redler LH, Ciccotti MG, Maffulli N, Longo UG, Bradley J (2013). Evaluation and management of hamstring injuries. Am J Sports Med.

[8] McGregor C, Ghosh S, Young DA, Maffulli N (2008). Traumatic and overuse injuries of the ischial origin of the hamstrings. Disabil Rehabil.

[9] Wood DG, Packham I, Trikha SP, Linklater J (2008). Avulsion of the proximal hamstring origin. J Bone Joint Surg Am.

[10] Chahal J, Bush-Joseph CA, Chow A, Zelazny A, Mather RC, Lin EC (2012). Clinical and magnetic resonance imaging outcomes after surgical repair of complete proximal hamstring ruptures: Does the tendon heal?. Am J Sports Med.

[11] Birmingham P, Muller M, Wickiewicz T, Cavanaugh J, Rodeo S, Warren R (2011). Functional outcome after repair of proximal hamstring avulsions. J Bone Joint Surg Am.

[12] Skaara HE, Moksnes H, Frihagen F, Stuge B (2013). Self-reported and performance-based functional outcomes after surgical repair of proximal hamstring avulsions. Am J Sports Med.

[13] Hofmann KJ, Paggi A, Connors D, Miller SL (2014). Complete avulsion of the proximal hamstring insertion: functional outcomes after nonsurgical treatment. J Bone Joint Surg Am.

[14] Shambaugh BC, Olsen JR, Lacerte E, Kellum E, Miller SL (2017). A comparison of nonoperative and operative treatment of complete proximal hamstring ruptures. Orthop J Sports Med.

[15] Pihl E, Jonsson KB, Berglöf M, Brodin N, Sköldenberg O, Hedbeck CJ (2021). Exploring the perth hamstring assessment tool and lower extremity functional scale in a proximal hamstring avulsion cohort: a cross-sectional study. Am J Sports Med.

[16] Fouasson-Chailloux A, Menu P, Mesland O, Dauty M (2020). Strength assessment after proximal hamstring rupture: a critical review and analysis. Clin Biomech (Bristol, Avon).

[17] Goutallier D, Postel JM, Bernageau J, Lavau L, Voisin MC (1994). Fatty muscle degeneration in cuff ruptures. Pre- and postoperative evaluation by CT scan. Clin Orthop Relat Res.

[18] Fuchs B, Weishaupt D, Zanetti M, Hodler J, Gerber C (1999). Fatty degeneration of the muscles of the rotator cuff: assessment by computed tomography versus magnetic resonance imaging. J Shoulder Elbow Surg.

[19] Heikkinen J, Lantto I, Piilonen J, Flinkkila T, Ohtonen P, Siira P (2017). Tendon length, calf muscle atrophy, and strength deficit after acute achilles tendon rupture: long-term follow-up of patients in a previous study. J Bone Joint Surg Am.

[20] Pons C, Borotikar B, Garetier M, Burdin V, Ben Salem D, Lempereur M (2018). Quantifying skeletal muscle volume and shape in humans using MRI: a systematic review of validity and reliability. PLoS ONE.

[21] van der Made AD, Peters RW, Verheul C, Smithuis FF, Reurink G, Moen MH (2022). Proximal hamstring tendon avulsions: comparable clinical outcomes of operative and non-operative treatment at 1-year follow-up using a shared decision-making model. Br J Sports Med.

[22] Pihl E, Skoldenberg O, Nasell H, Jonhagen S, Kelly Pettersson P, Hedbeck CJ (2019). Patient-reported outcomes after surgical and non-surgical treatment of proximal hamstring avulsions in middle-aged patients. BMJ Open Sport Exerc Med.

[23] Harris PA, Taylor R, Thielke R, Payne J, Gonzalez N, Conde JG (2009). Research electronic data capture (REDCap)–a metadata-driven methodology and workflow process for providing translational research informatics support. J Biomed Inform.

[24] Yushkevich PA, Piven J, Hazlett HC, Smith RG, Ho S, Gee JC (2006). User-guided 3D active contour segmentation of anatomical structures: significantly improved efficiency and reliability. Neuroimage.

[25] Mukaka MM (2012). Statistics corner: a guide to appropriate use of correlation coefficient in medical research. Malawi Med J.

[26] Nakamura Y, Yokoya S, Harada Y, Shiraishi K, Adachi N, Ochi M (2017). The prospective evaluation of changes in fatty infiltration and shoulder strength in nonsurgically treated rotator cuff tears. J Orthop Sci.

[27] Schmidt CC, Diaz VA, Weir DM, Latona CR, Miller MC (2012). Repaired distal biceps magnetic resonance imaging anatomy compared with outcome. J Shoulder Elbow Surg.

[28] Youn SM, Rhee YG, Rhee SM (2021). Nontendinous healing after repairing of retracted rotator cuff tear: an imaging study. J Shoulder Elbow Surg.

[29] Lansdown DA, Lee S, Sam C, Krug R, Feeley BT, Ma CB (2017). A prospective, quantitative evaluation of fatty infiltration before and after rotator cuff repair. Orthop J Sports Med.

[30] Silder A, Heiderscheit BC, Thelen DG, Enright T, Tuite MJ (2008). MR observations of long-term musculotendon remodeling following a hamstring strain injury. Skeletal Radiol.

[31] Evangelidis PE, Massey GJ, Pain MTG, Folland JP (2015). Biceps femoris aponeurosis size: A potential risk factor for strain injury?. Med Sci Sports Exerc.

[32] Schiefer M, Mendonça R, Magnanini MM, Fontenelle C, Pires Carvalho AC, Almeida M (2015). Intraobserver and interobserver agreement of Goutallier classification applied to magnetic resonance images. J Shoulder Elbow Surg.

[33] Barnouin Y, Butler-Browne G, Voit T, Reversat D, Azzabou N, Leroux G (2014). Manual segmentation of individual muscles of the quadriceps femoris using MRI: a reappraisal. J Magn Reson Imaging.

[34] Dixon WT (1984). Simple proton spectroscopic imaging. Radiology.

